# Noninvasive Imaging of Human Atrial Activation during Atrial Flutter and Normal Rhythm from Body Surface Potential Maps

**DOI:** 10.1371/journal.pone.0163445

**Published:** 2016-10-05

**Authors:** Zhaoye Zhou, Qi Jin, Long Yu, Liqun Wu, Bin He

**Affiliations:** 1 Department of Biomedical Engineering, University of Minnesota, Minneapolis, Minnesota, United States of America; 2 Department of Cardiology, Ruijin Hospital Affiliated to Shanghai Jiaotong University School of Medicine, Shanghai, China; 3 Institute for Engineering in Medicine, University of Minnesota, Minneapolis, Minnesota, United States of America; Loyola University Chicago, UNITED STATES

## Abstract

**Background:**

Knowledge of atrial electrophysiological properties is crucial for clinical intervention of atrial arrhythmias and the investigation of the underlying mechanism. This study aims to evaluate the feasibility of a novel noninvasive cardiac electrical imaging technique in imaging bi-atrial activation sequences from body surface potential maps (BSPMs).

**Methods:**

The study includes 7 subjects, with 3 atrial flutter patients, and 4 healthy subjects with normal atrial activations. The subject-specific heart-torso geometries were obtained from MRI/CT images. The equivalent current densities were reconstructed from 208-channel BSPMs by solving the inverse problem using individual heart-torso geometry models. The activation times were estimated from the time instant corresponding to the highest peak in the time course of the equivalent current densities. To evaluate the performance, a total of 32 cycles of atrial flutter were analyzed. The imaged activation maps obtained from single beats were compared with the average maps and the activation maps measured from CARTO, by using correlation coefficient (CC) and relative error (RE).

**Results:**

The cardiac electrical imaging technique is capable of imaging both focal and reentrant activations. The imaged activation maps for normal atrial activations are consistent with findings from isolated human hearts. Activation maps for isthmus-dependent counterclockwise reentry were reconstructed on three patients with typical atrial flutter. The method was capable of imaging macro counterclockwise reentrant loop in the right atrium and showed inter-atria electrical conduction through coronary sinus. The imaged activation sequences obtained from single beats showed good correlation with both the average activation maps (CC = 0.91±0.03, RE = 0.29±0.05) and the clinical endocardial findings using CARTO (CC = 0.70±0.04, RE = 0.42±0.05).

**Conclusions:**

The noninvasive cardiac electrical imaging technique is able to reconstruct complex atrial reentrant activations and focal activation patterns in good consistency with clinical electrophysiological mapping. It offers the potential to assist in radio-frequency ablation of atrial arrhythmia and help defining the underlying arrhythmic mechanism.

## Introduction

Electrophysiological mapping is an important tool for the management of cardiac arrhythmias. Information for electrical substrate and ectopic foci can aid in the individualization of therapeutic plan and allows effective guidance of catheter ablation. However, the catheter-based minimal invasive procedure might increase patients’ burden due to the requirement of sedation and the need of prolonged time for acquiring high-resolution bi-chamber maps. Instead, cardiac electric source imaging is an alternative way to delineate the electrophysiology of the heart. Such approach translates the body surface ECG distribution into cardiac electric sources throughout the myocardial domain, thus allowing direct interpretation of electrical activities. By reconstructing bi-chamber cardiac electric activities with a single heartbeat, it allows effective guidance of ablation for arrhythmias with unstable hemodynamics and further facilitates understanding of the mechanisms of complex arrhythmias.

Many efforts have been made in the development of cardiac electric source imaging techniques. Investigations have been made to reconstruct the single moving dipole [1–3], the epicardial potentials [4–7], and the heart surface isochrones [8–14], by solving the electrocardiographic inverse problem. Various forward cardiac models have been developed to understand cardiac electrophysiology using computational approaches [15, 16]. Recently, efforts have been made to image the cardiac electrical activities throughout the 3-dimensional (3D) myocardium [17–29]. As atrial arrhythmias account for a large patient population, many efforts have been made to noninvasively image atrial electrical activity by using the above-mentioned approaches. In the last two decades, the cardiac electrical imaging technique has been mainly utilized to image atrial activations with a focal-onset or a macro-reentry mechanism [12, 13, 30–39]. In these investigations, validations on human subjects were performed by quantitatively comparing noninvasively-obtained activation sequences in the atrium with the electroanatomic maps during atrial pacing, evaluating the performance of identifying chambers of focal origins or reentry, or qualitatively comparing flutter circuits with CARTO map. Although these studies have contributed to the management of atrial arrhythmias, rigorous and quantitative validations is needed, specifically, for those patients with macro-reentry in the atrium, to fully demonstrate the clinical validity of the technique.

In recent years, a novel 3D cardiac electrical imaging (CEI) approach has been developed to mathematically model the ventricular electrical activities by using equivalent current density (ECD) distribution. The method has been rigorously validated with systematic computer simulations and animal studies using 3D intracardiac mapping [24, 40–42]. Due to the fact that atrial electrical signal is much smaller than the ventricular signal, imaging the atrial activities can be intrinsically challenging. The purpose of the present study is to further extend the application of the CEI technique from imaging ventricular activation to imaging atrial activation, in the case of both focal pattern and reentrant excitation in the atria, in a quantitative manner. The performance was quantitatively assessed by comparison with invasive mapping using CARTO. The imaging results from normal subjects and patients with atrial flutter were consistent with both CARTO maps and literatures, suggesting that CEI is capable of delineating atrial activation pattern on a single-beat basis and that it is feasible to reconstruct reentrant activation with close similarity to direct endocardial mapping.

## Materials and Methods

**Fig 1:**
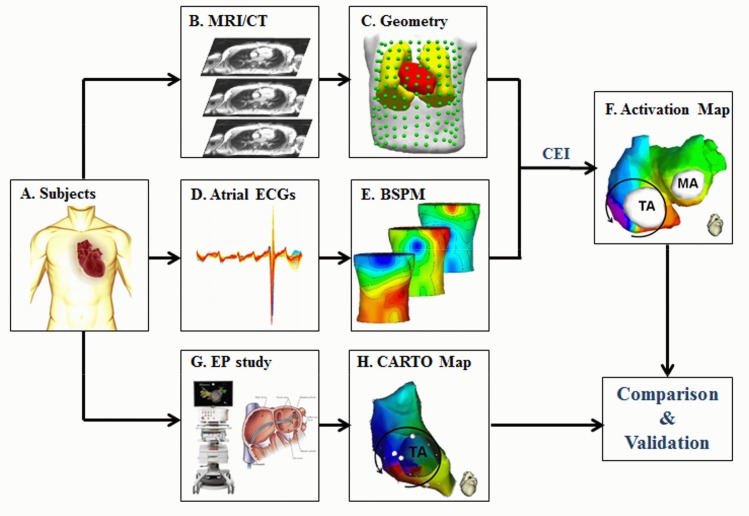
Schematic diagram of the present study. BSPM = Body surface potential map.

Fig 1 shows the schematic diagram of the study design. BSPMs were obtained in all subjects. CT and MRI were performed in patients with atrial flutters (AFL) and healthy subjects, respectively. Atrial activation maps were imaged from BSPM with the aid of geometry heart-torso models, and compared with CARTO maps when available in the patients.

### Subjects

Seven subjects (5 males; 2 females) with atrial flutter (AFL, n = 3) or sinus rhythm (n = 4) were recruited to participate in the present study. The characteristics of the subjects are summarized in Table 1. All protocols were approved by the Institutional Review Board (IRB) of the Ruijin Hospital (affiliated to Shanghai Jiao Tong University School of Medicine, Shanghai, China) and the University of Minnesota, where written informed consent was obtained from all subjects before data collection was performed.

**Table 1:** Clinical characteristics of the study population. M = Male; F = Female; LA = Left atrium; RA = Right atrium; TI = Tricuspid isthmus; SR = Sinus rhythm.

Subject #	Age	Gender	Classification	Clinical History*	Atrial Size	Clinical intervention	Clinical Outcome
AL2	60	M	AFL	N/A	Normal	Linear ablation at TI	SR
AY3	67	F	AFL	Pulmonary Hypertension Tricuspid Insufficiency	LA Enlargement	Linear ablation at TI	SR
AX4	47	F	AFL	Hypertension Aortic Insufficiency Mitral Insufficiency	LA Enlargement	Linear ablation at TI	SR
NS5	36	M	SR	N/A	Normal	N/A	N/A
NS6	32	M	SR	N/A	Normal	N/A	N/A
NS7	26	M	SR	N/A	Normal	N/A	N/A
NS8	34	M	SR	N/A	Normal	N/A	N/A

*Selected medical history.

### Anatomic data collection

Computer tomography (CT) was performed on patients with AFL in order to obtain the heart-torso geometries. The heart geometries were obtained by continuous volume scanning from the great vessel level down to the diaphragm with intravenous (IV) contrast. The slice thickness was 0.4 mm and was fine enough for the segmentation of a refined heart model. Additional torso scans were performed with a slice thickness of 6 mm from the level of collar bone down to the lower abdomen, which were used to build the thorax model. The in-plane resolution of the CT scans was fixed at 512 x 512 pixels. Respiration was held by alerting the patient before scanning in order to avoid respiratory artifact. Continuous ECG was monitored and used for gating the CT scanner. Magnetic resonance imaging (MRI) was performed on the other four healthy subjects with the same geometric coverage of CT. The slice thickness of MRI was 1–2 mm for the heart and 5 mm for the torso.

The CT and MRI images were further processed by commercial software (Curry 6.0, Neuroscan, North Carolina) to build the individual heart-torso geometry models. The heart and torso images were coupled based on important cardiac anatomical landmarks, such as the apex, and the co-registration errors were minimized with the assistance of Curry 6.0. Detailed anatomy structures including the atria, the ventricles, the lung and the torso were segmented. For the segmentation of the atria, important anatomical structures, like the superior vena cava (SVC), inferior vena cava (IVC), tricuspid annulus (TA) and mitral annulus (MA), were identified and segmented.

### Body surface potential mapping

For each patient, the BSPMs were recorded at resting state (supine position with smooth breath) by using a total of 208 channels of Ag-AgCl carbon electrodes (BioSemi Active-Two), with a sampling rate of 2,048 Hz and a 24 bit analog-to-digital converter. In order to retrieve comprehensive electrical activities over the torso, the electrodes were placed on both the anterior chest (n = 144) and the posterior trunk (n = 64). The surface ECGs were further filtered with a 1–400 Hz bandpass filter, followed by a 50-Hz notch filter for AFL patients and a 60-Hz notch filter for normal subjects to remove the utility frequency component. The Wilson central terminal (WCT) was used as the reference of the surface ECGs. After the BSPM recording, the locations of the electrodes were recorded using a radio frequency digitizer (Fastrak, Polhemus Inc., Vermont). During the recording of BPSM, subjects were told to keep still with smooth respiration in order to minimize baseline wandering and motion artifacts.

### Data analysis

The physical-model-based CEI technique was previously used to reconstruct the activation sequence throughout the ventricular myocardium and validated with animal studies [24, 40–42]. In the present study, the application of CEI was extended to estimate atrial activation sequences for the first time. The forward computation and inverse method were described in details in previous publications [24, 40, 41]. Briefly, geometry models were reconstructed from MRI/CT and translated into boundary element models (BEM), using a commercial software package Curry 6.0 (Neuroscan, North Carolina). Due to the thinness of atrial wall, the electrical activities were assumed to be distributed over 2-dimensional (2-D) surface other than the 3-D volume, hereby the source surfaces are defined as the endocardium of the left atrium (LA) and right atrium (RA). The LA and RA were discretized into 1445±242 grid points with a spatial resolution of 3mm for the inverse computation. The 2-D distributed ECD representing cardiac electric sources were thus reconstructed by coupling measured BSPM with BEM to solve the inverse problem.

### Principle of current density reconstruction

In the present study, the cardiac source was modeled as the ECD distributed over the endocardial surfaces of the LA and RA. The ECD distribution was then reconstructed from the BSPM by solving the so-called inverse problem. The linear forward model relating cardiac sources to BSPM can be expressed as follows:

Φ(t)=LJ(t) (1)

Where Φ(*t*) is a M × 1 column vector of body surface potentials measured from M body surface electrodes at time t, *J*(*t*) is a 3N × 1 column vector of ECD distribution from N myocardial grid points, and *L* is the M × 3N source-to-field transfer matrix relating ECD distribution to the body surface potentials. This linear inverse problem was solved using the minimum norm least square (MNLS) solution [24, 43, 44], which minimizes the following objective function:

$$\underset{\text{J}(\text{t})}{\text{min}}({\left\|\Phi (t)-LJ(t)\right\|}_{2}^{2}+\lambda {\left\|WJ(t)\right\|}_{2}^{2})$$ (2)

Where ${\left\|•\right\|}_{2}^{2}$ symbolizes the squared Euclidean norm, *W* is a diagonal location weighting matrix to remove depth bias, and *λ* is the regularization parameter [45]. The MNLS method [44] has been widely used to solve Eq (1) and was also used in the present study to provide optimal estimate of ECD in the least-squares sense. The dipole sources *J*(*t*) are assumed to have arbitrary orientations and strengths but fixed and pre-assigned locations. Therefore the lead field matrix L remains unchanged. The model term ${\left\|WJ(t)\right\|}_{2}^{2}$ is needed for searching unique solution in a problem with such large number of free parameters (e.g. unknown dipole moment per location) [17, 46]

By definition, ECD is proportional to the gradient of transmembrane potentials. From the peak criterion in our previous studies [24], the activation time *τ* at a given location *i* was determined as the time instant corresponding to the maximum value of ECD waveform *J*(*i*,*t*):

$$\tau (i)=\begin{array}{c} \arg\;\max\\ t\in T \end{array}(|J(i,t)|)$$ (3)

Once the current density *J*(*i*,*t*) is reconstructed, the activation time *τ*(*i*) at *i*^*th*^ grid point can be determined by Eq (3).

In order to evaluate the performance of the CEI technique in imaging atrial activation, the endocardial activations of AFL patients were obtained from electroanatomic maps (CARTO, Biosense-webster) and compared with imaged activation times quantitatively by computing the correlation coefficients (CC) and relative error (RE). The co-registration between CARTO anatomic map and CT geometry was optimized by minimizing the average distance between the two sets of points on the surfaces of the anatomic geometries [25]. For patients with CARTO maps, quantitative comparisons were performed if the average geometry co-registration difference between CT and CARTO is smaller than 1 cm after optimization. A total of 32 cycles of AFL were analyzed, and the imaged activation sequences from single-beat estimations were compared with the average activation sequences.

## Results

### Normal atrial activation

**Fig 2:**
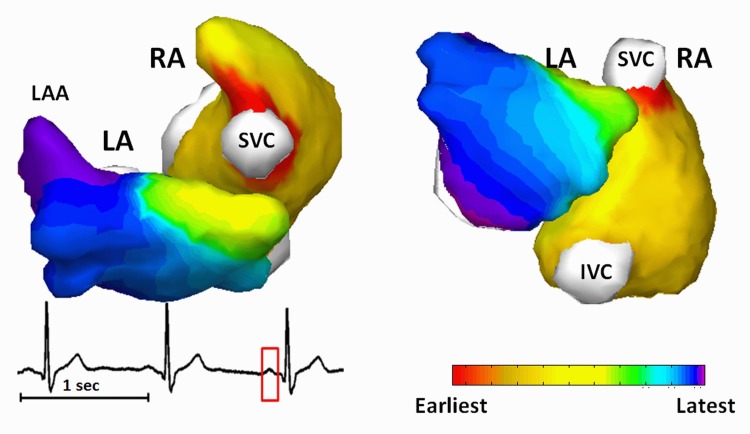
Imaged normal atrial activation from subject NS5. IVC = Inferior vena cava; LA = Left atrium; LAA = Left atrial appendage; RA = Right atrium; SVC = Superior vena cava.

Normal atrial activations were reconstructed from 4 healthy subjects. BSPMs during P-wave were coupled with heart-torso geometry to image the atrial activation sequence. Fig 2 shows one representative example of normal atrial activation obtained from a 36-years old healthy male subject. The activation sequence is color coded from red to violet, corresponding to the earliest and the latest activation. The P-wave used for reconstruction is marked with red window from lead II. The earliest activation initiated in RA from the right border of SVC, which is corresponding to the anatomic location of sinoatrial (SA) node (Fig 2, red area in RA). For LA excitation, the electrical impulse passed to LA through Bachman’s bundle, as indicated by the yellow region in the LA of Fig 2. The latest atrial excitation was found at the inferior LA and LA appendage (LAA).

**Fig 3:**
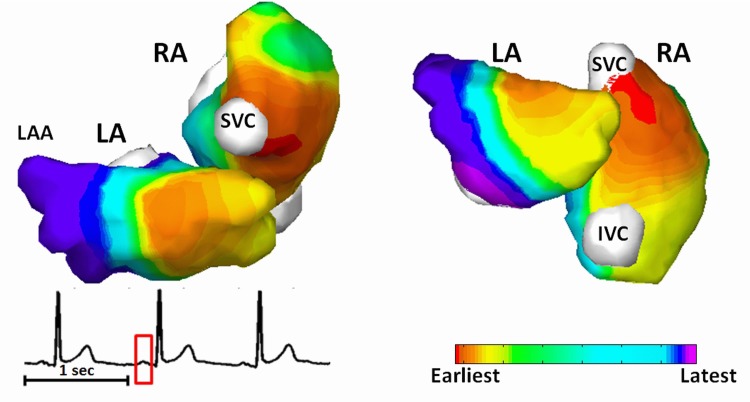
Imaged normal atrial activation from subject NS7. IVC = Inferior vena cava; LA = left atrium; LAA = Left atrial appendage; RA = right atrium; SVC = Superior vena cava.

Fig 3 shows another example of the normal atrial activation from a 26-years old male. The earliest activation starts from the SA node located at inferior and right area of SVC in RA (Fig 3, red area in RA), travels to LA through Bachman’s bundle (Fig 3, light-red area in LA), and ends up at the inferior LA (Fig 3, violet area). The locations of SA node were identified from all subjects at the regions around SVC. For the group of healthy subjects, the atrial activation patterns show intra-group similarity, which can also be observed by comparing Figs 2 and 3. Furthermore, the features of normal atrial excitation, e.g. SA nodes in the RA which is indicated by earliest activation sites, the anatomical location of Bachman bundle as indicated by earliest excitation site in LA, and the latest activation sites in LA, are in good agreement with direct recordings from isolated human hearts in previous study [47].

### Atrial flutter

**Fig 4:**
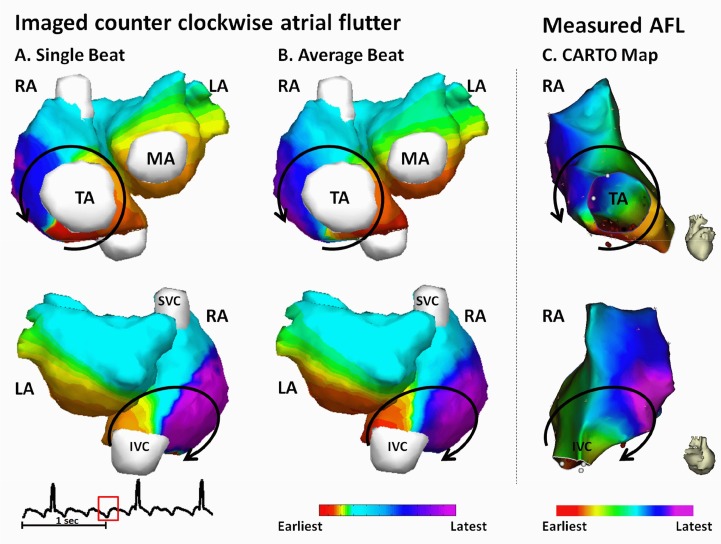
Activation maps for patient AX4. (A) Noninvasively imaged AFL reentry circuit from one single beat. Solid black lines represent propagation wavefronts in RA. (B) Average AFL reentry circuit. (C) CARTO activation map. AFL = Atrial flutter; IVC = Inferior vena cava; LA = Left atrium; MA = Mitral annulus; RA = Right atrium; SVC = Superior vena cava; TA = Tricuspid annulus.

Fig 4 shows the feasibility of CEI to image AFL in patients. This is a 47-years old female patient with 9-years’ history of atrial flutter who did not response to anti-arrhythmia medications. The surface ECG is characterized by saw-tooth waveform at a rate of 285 beats/min with a cycle length of 210 ms. Fig 4A and 4B depict one cycle of imaged counter-clockwise typical AFL and the average AFL activation, respectively. The reentry circle in the RA is marked in solid black line. The imaged reentrant circuit ascends from the tricuspid isthmus (TI) near the coronary sinus ostium (CSO), forming an upward wavefront traveling through the septum. The wavefront is then split into two branches traveling around SVC anteriorly and posteriorly: one propagates anteriorly into the area between the SVC and the superior TA; the other excitation wavefront travels posteriorly between SVC and IVC. The two wavefronts then join together and descend along the RA free-wall, and finally reach the isthmus between TA and IVC at the end of the reentry circuit. The LA activation does not involve in the reentry circuit. The earliest excitation in the LA starts from the inferior LA, indicating the trans-septal activation from RA through coronary sinus (CS). The excitation then propagates upward along the anterior and posterior LA wall. The single-beat-based imaging result and the average activation sequence share similar global activation pattern, which are featured by the macro reentry in the RA, the intra-atria conduction through CS, and the inferior-to-superior excitation in the LA (Fig 4A and 4B). Quantitative comparison between the two activation sequences yield a CC of 0.94 and a RE of 0.37.

The patient received ablation therapy and the AFL was terminated with linear ablation in the isthmus between the IVC and TA (Fig 4C, red balls). Endocardial activation map was acquired by using CARTO in EP study (Fig 4C) during stable AFL rhythm. The global activation pattern of noninvasively imaged AFL was found to be in good agreement with the electroanatomic map, which is reflected by a CC of 0.77 and a RE of 0.28.

For the patient, a total of 12 independent cycles of atrial flutter were analyzed. The activation sequences imaged from singles beats were quantitatively compared with the average activation sequence as well as the CARTO map. Overall, the activation sequences obtained from independent beats, the average activation sequence, and the activation sequence generated by CARTO share similar global activation pattern and are also in good quantitative agreement with each other (Table 2, subject AX4).

**Table 2:** Quantitative evaluations using the mean activation sequence and CARTO as the references. CC = Correlation coefficient; RE = Relative error.

Reference	Subject #	Number of Beats	CC	RE
Mean Activation Sequence	AL2	10	0.90±0.04	0.30±0.06
	AY3	10	0.90±0.03	0.29±0.05
	AX4	12	0.92±0.03	0.27±0.05
CARTO	AX4	12	0.70±0.04	0.42±0.05

**Fig 5:**
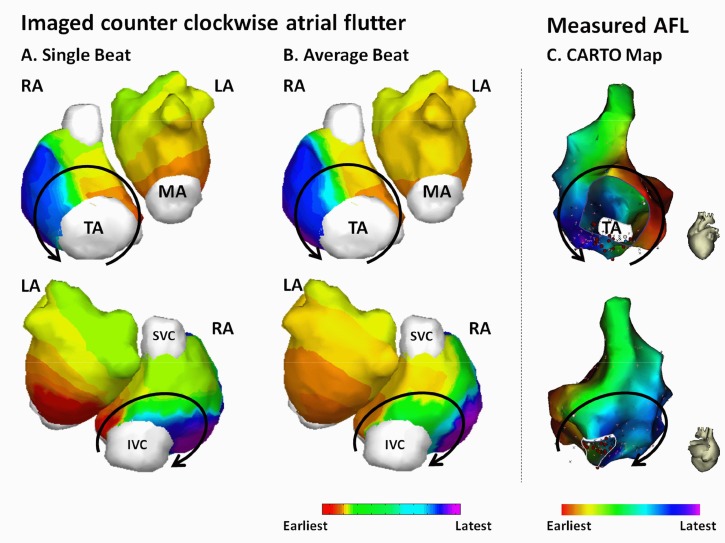
Activation maps for patient AL2. (A) Noninvasively imaged AFL reentry circuit from one single beat. Solid black lines represent propagation wavefronts in RA. (B) Average AFL reentry circuit. (C) CARTO activation map. AFL = Atrial flutter; IVC = Inferior vena cava; LA = Left atrium; MA = Mitral annulus; RA = Right atrium; SVC = Superior vena cava; TA = Tricuspid annulus.

Fig 5A shows another example of the reentry circuit reconstructed from a 60-years old male. Like most counterclockwise atrial flutter, the activation map is featured by macro reentry in the RA circulating around the tricuspid valve annulus and RA-to-LA propagation through CS (earliest excitation in LA observed at the inferior region near MA). The CC between the single-beat-based activation sequence and the average activation sequence (Fig 5B) is 0.90, and the RE is 0.30. For this patient, a total of 10 independent cycles of atrial flutter were analyzed. On average, the single-beat-based global activation sequences share 90% of similarity with the average activation sequence (Table 2, subject AY3).

The patient received ablation therapy and the electroanatomic map was acquired by using CARTO in electrophysiological (EP) study (Fig 5C) during stable AFL rhythm. The ablation was delivered at the isthmus between IVC and TA, as shown by the red balls in Fig 5C. For this patient, Fig 5 has demonstrated that, both the imaged reentry circuit from single beat and the average reentry circuit are in good agreement with the measurements from clinical EP study.

For AFL, a total of 32 beats were analyzed. For each patient, the single-beat-based activation sequences were averaged to produce the subject-specific mean activation sequence, which was used as a reference to quantitatively evaluate the performance of CEI. Overall, the mean CC is 0.91±0.03, and the mean RE is 0.29±0.05. For each patient, the CC and RE between single-beat-based activation sequences and the mean activation sequences are summarized and reported in Table 2.

## Discussion

As cardiac arrhythmia remains a major public health problem, the study of cardiac electrophysiology on individual patients is important for personalizing disease management and treatment. Various approaches have been pursued to detect and analyze atrial electrical activity [48, 49]. CEI translates the body surface electrical potentials into cardiac sources and provides a noninvasive approach to image cardiac activation [50–54]. Such noninvasive approach may leverage clinical treatment by assisting in planning for the intervention strategy, localizing ablation targets pre-surgically to shorten ablation time, and helping with post-surgery evaluation over time. In addition, considering the existing difference between animal models and human subjects, it may also facilitate understanding to electrophysiological characteristics and mechanisms of spontaneous arrhythmias in intact human hearts.

For the development of cardiac electrophysiological imaging technology, validations on human subjects are important to demonstrate the clinical validity and establish its clinical value. In previous studies of atrial electrical imaging on human subjects, quantitative evaluations were reported for activations with focal onset [12, 30–37, 39], by using CARTO recording as the reference. In contrast, the present study has quantitatively evaluated the performance of imaging atrial macro-reentry, by comparing the noninvasively obtained reentry circuits with the CARTO maps. Such quantitative assessment constitutes an important part of the clinical study for the technique development. Furthermore, multiple beats (> = 10) from each individual were analyzed and the intra-subject similarities of the imaged activations were evaluated. The high correlations between single-beat-based activation sequences and the average activation sequences (CC = 0.91±0.03) further indicate the reliability of the technique in clinical application. These statistical outcomes have shown the reproducibility of atrial activation imaging on different subjects.

The present study shows that CEI is able to image atrial activations with both focal pattern and reentrant pattern. Previous studies on animal models demonstrated that CEI can reconstruct ventricular activation with a localization error of 7 mm in canines with pacing rhythm and ventricular tachycardia [41]. In the present study, we further evaluated the performance of CEI on imaging atrial activations in a clinical setting, and compared the results with clinical EP studies from the same patients, with the averaged activation maps, and with literatures from invasive measurements. The results from normal subjects (Figs 2 and 3) demonstrated the capability of CEI of imaging focal atrial activations. The activation details regarding initiation of activation wavefront, conducting pathway of Bachman bundle, latest activation sites like LAA and the inferior LA, were identified by the CEI. The imaged activation patterns are consistent with the findings on isolated human hearts [47], indicating that CEI has the potential to localize ectopic foci and image global activation maps not only in the ventricles [23, 40–42] but also in the atria. For AFL patients, CEI reconstructed reentry details that could not be observed directly from ECG or BSPM. The imaged reentry was defined in details within the RA (Figs 4 and 5) and in good agreement with the EP measurements, which is reflected by an average of CC = 0.70±0.04 and an average RE of 0.42±0.05. Besides the macro-reentry in the RA, CEI was also able to capture the electrical conduction from RA to LA through coronary sinus and the inferior-to-superior activation in the LA. Our results suggest that CEI can reliably reconstruct both focal and reentry atrial activation patterns and its application is not limited to ventricles.

In the present study, the CEI technique was utilized to image normal atrial activation and atrial reentrant circuits for the first time. Our results show the feasibility to image atrial activation with focal as well as reentrant patterns with close match to literatures and good agreement with clinical EP findings. While the present results are promising, this is an open trial study and the number of subjects is relatively small. With the feasibility demonstrated in the present work, future investigation should be conducted with a double-blinded study design in a large number of subjects with atrial arrhythmias, including age/gender matched healthy controls, to establish the clinical values of using the present technique for clinical diagnosis and aiding catheter ablation in a clinical setting. With more rigorous quantitative validation studies and further development in the future, the CEI may become a complementary tool in clinical practice aiding diagnosis and ablation planning (e.g., localizing ectopic foci and the critical zones for ablation), and further assist in investigation of arrhythmic mechanism.

## Conclusions

We have investigated noninvasive imaging of atrial activation sequence using the cardiac electric imaging approach. Our results in seven human subjects including healthy subjects and patients with atrial flutter suggest that the cardiac electric imaging is capable of delineating both focal and reentrant mechanisms in good consistency with electrophysiological findings and literatures. The cardiac electric imaging promises to offer a noninvasive tool to define arrhythmic mechanism and guide catheter ablation.
